# Nonconvulsive status epilepticus following cardiac arrest: overlooked, untreated and misjudged

**DOI:** 10.1007/s00415-022-11368-5

**Published:** 2022-09-08

**Authors:** Pia De Stefano, Peter W. Kaplan, Hervé Quintard, Margitta Seeck, Raoul Sutter

**Affiliations:** 1 Neuro-Intensive Care Unit, Department of Intensive Care, University Hospital of Geneva, Geneva, Switzerland; 2 EEG and Epilepsy Unit, Neurology Unit, Department of Clinical Neurosciences and Faculty of Medicine of Geneva, University Hospital of Geneva, Geneva, Switzerland; 3 Department of Neurology, Johns Hopkins Bayview Medical Center, Baltimore, MD USA; 4 Department of Intensive Care, University Hospital Basel, Basel, Switzerland; 5 Division of Neurophysiology, Department of Neurology, University Hospital Basel, Basel, Switzerland; 6 Medical Faculty, University of Basel, Basel, Switzerland; 7 Department of Clinical Research, University of Basel, Basel, Switzerland

**Keywords:** Non-convulsive status epilepticus, NCSE, Status epilepticus, Cardiac arrest, Ischemic-hypoxic encephalopathy

## Abstract

**Aims:**

Seizures and status epilepticus (SE) are detected in almost a third of the comatose cardiac arrest survivors. As the literature is quite exhaustive regarding SE with motor symptoms in those patients, little is known about nonconvulsive SE (NCSE). Our aim was to compile the evidence from the literature of the frequency and outcome of NCSE in adult patients remaining in coma after resuscitation.

**Methods:**

The medical search PubMed was screened for most relevant articles reporting the emergence and outcome of NCSE in comatose post-resuscitated adult patients.

**Results:**

We identified 11 cohort studies (four prospective observational, seven retrospective) including 1092 patients with SE in 29–96% and NCSE reported in 1–20%. EEG evaluation started at a median of 9.5 h (range 7.5–14.8) after cardiac arrest, during sedation and targeted temperature management (TTM). Favorable outcome after NCSE occurred in 24.5%. We found no study reporting EEG to detect or exclude NCSE in patients remaining in coma prior to the initiation of TTM and without sedation withing the first hours after ROSC.

**Discussion:**

Studies on NCSE after ROSC are scarce and unsystematic, reporting favorable outcome in every fourth patient experiencing NCSE after ROSC. This suggests that NCSE is often overlooked and outcome after NCSE is not always poor. The low data quality does not allow firm conclusions regarding the effects of NCSE on outcome calling for further investigation. In the meantime, clinicians should avoid equating NCSE after ROSC with poor prognosis.

## Introduction

Cardiac arrest (CA) is the most common cause of death, resulting in approximately 350,000 deaths annually in out-of-hospital CAs in the Unites States [1]. The degree of hypoxic-ischemic brain injury varies in its clinical manifestation, ranging from mild and transient forms of encephalopathy to deep coma and brain death [2–4].

For patients with persistent coma following return of spontaneous circulation (ROSC), the recommendations for initial care from the European Resuscitation Council (ERC) and the European Society for Intensive Care Medicine (ESICM) include artificial ventilation, sedation, and neuroprotective measures including continuous monitoring of core temperature to detect and suppress fever (defined as a temperature > 37.7 °C) for at least 72 h [5–7]. Hypothermia and targeted temperature management (TTM) are in fact not recommended anymore [6].

In almost a third of these patients, seizures and status epilepticus (SE) are detected with or without clinically overt signs and symptoms [8–13]. As the literature is quite exhaustive regarding SE with motor symptoms in comatose CA-survivors, little is known about nonconvulsive SE (NCSE) in this context. While recovery from SE post cardiac arrest has been described with good outcome in selected cases [9, 11, 14–16], it has been an independent predictor of poor prognosis in larger cohorts [8], especially with seizure-associated motor symptoms [8, 17, 18]. SE with motor symptoms are reported more frequently than NCSE [9, 19]. To what degree these proportions are influenced by detection and/or reporting biases remains unknown, but is likely to be the case as the lack of overt clinical signs or subtle presentation of NCSE can be missed without continuous electroencephalography (c-EEG) [20]. A recent trial found no benefits of aggressive treatment regarding outcome compared to standard care in post-CA comatose patients showing rhythmic and periodic discharges of any frequency on EEG (without limiting the study to EEG patterns reflecting SE [21]) performed within a median of 13.5 h following ROSC [22]. However, it seems plausible that unrecognized and untreated NCSE may cause subsequent neurologic injury and worsen outcome. This may be relevant especially in patients without additional early markers of an unfavorable outcome, such as age > 65 years, unshockable rhythm, and no conversion to shockable rhythms during resuscitation [23–25].

We aimed to compile the evidence from the literature of the frequency and outcome of NCSE in adult patients remaining in coma after resuscitation.

## Methods

The most recent publications since 2000 reporting neurologic workups in patients with persistent coma following ROSC were screened regarding frequency and outcome of status epilepticus post-CA and the type or the semiology of post-hypoxic-ischemic SE. Since SE can be defined either as “with” or “without prominent motor symptoms” (i.e., NCSE meaning, electrographic status epilepticus in coma) [26], we considered that patients not explicitly described as having motor symptoms, myoclonic or convulsive SE had NCSE. Data regarding study design, number of patients, time of EEG performance from CA, type of EEG performed, number of patients with SE, clinical signs of SE, proportion of patients with NCSE, reported outcomes of patients with SE and NCSE, presence of targeted temperature or sedation management and outcomes defined according to the Cerebral Performance Category (CPC) [27] were collected. Cerebral Performance Category (CPC) score is a five point scale that ranges from good cerebral performance (1), moderate cerebral disability (2), severe cerebral disability (3), coma or vegetative state (4) to brain death (5), and that can be commonly dichotomized into “good” (CPC 1–2 or CPC 1–3) versus “poor” (CPC 3–5 or CPC 4–5) outcome when used as a primary or secondary outcome in research clinical studies [28].

We considered favorable outcome a CPC score of 1–2 at discharge and unfavorable outcome a CPC ≥ 3. In case, the exact number of CPC1–2 patients was not reported, and patients were described as having CPC ≤ 3, we did not consider them as favorable outcome.

Since the new version of the American Clinical Neurophysiology Society’s Standardized Critical Care Terminology [29] has been published in 2021, we could not apply those criteria for SE to our screening; nevertheless we do not believe this represents a major methodological issue since consensus on the criteria and definition of SE and especially NCSE was already established by the International League Against Epilepsy [26], as outlined by the Salzburg criteria in 2015 [30, 31].

## Results

Our review of the literature identified 11 studies (four prospective observational, seven retrospective) including 1092 patients reporting SE in 29–96% with NCSE in 1–20% during sedation and TTM. We could not identify any studies reporting EEG monitoring to detect or exclude NCSE in resuscitation adult patients remaining in coma prior to the TTM and without sedation within the first hours (i.e., < 7.5 h) after ROSC.

**Fig. 1:**
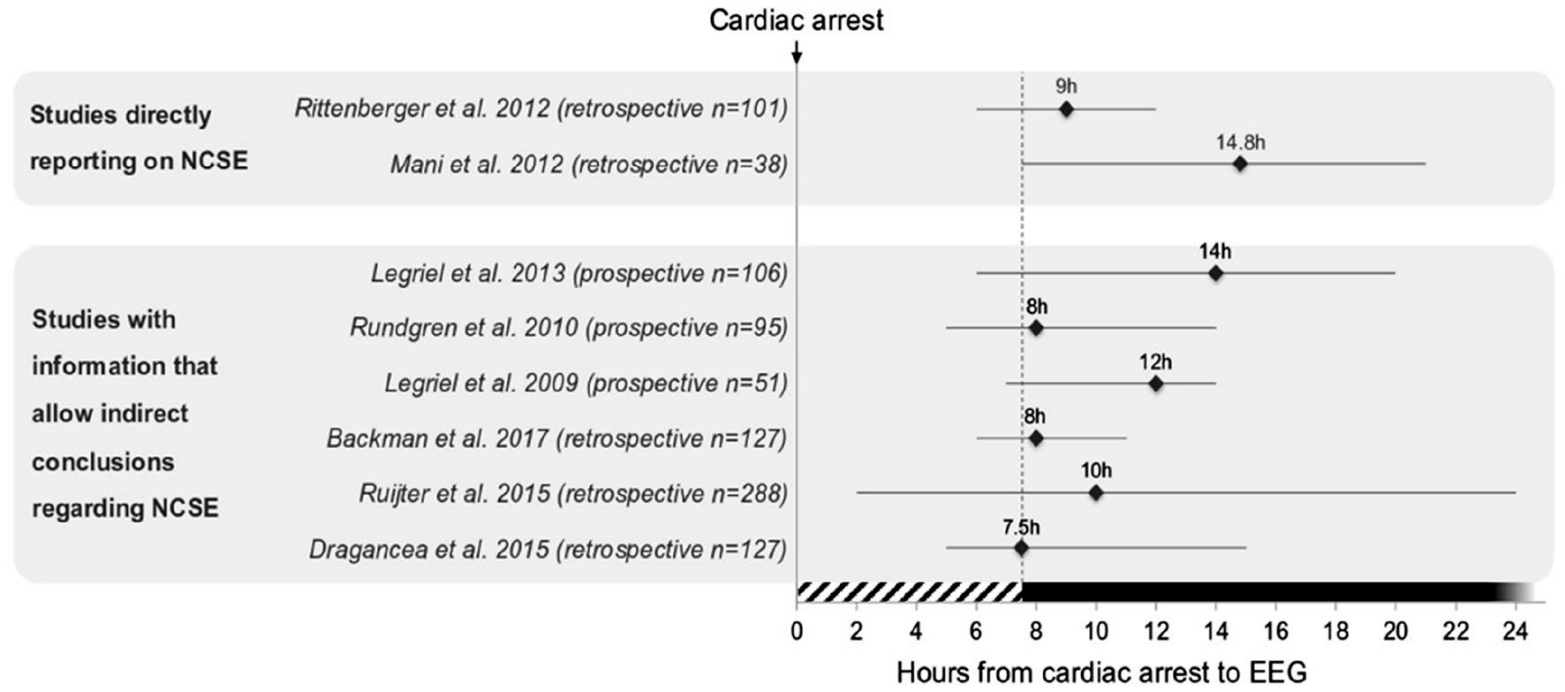
Reported median time (and range) from cardiac arrest to EEG from the literature. *NCSE* nonconvulsive status epilepticus

When considering only patients showing a CPC score 1–2, NCSE was associated with favorable outcome in 12 of 49 patients (24.5%),

Detailed information of studies describing or indirectly reporting NCSE without a systematic EEG driven screening for NCSE are compiled in Table 1. The median time from CA to performance of first EEG of eight studies providing such information is presented in Fig. 1. The overall median from CA to EEG was 9.5 h (range 7.5–14.8 h).

**Table 1:** Studies describing or indirectly reporting the emergence and frequency of NCSE within the first hours after ROSC

Number of patients	Total SE		Outcome of total SE	NCSE			Sedation and TTM	Outcome of NCSE	EEG type	Paper	Study type
	*N*	%		*N*	% of post-CA patients	% of SE patients					
*Studies directly reporting on NCSE*											
74 (prosp. cohort)	28	38%	1 CPC 2 2 CPC 2–3 25 CPC 5	4 (described)	5%	14%	Sedation TTM (33 °C)	1 CPC 2 1 MCS 2 CPC 5	s-EEG	Rossetti et al. [9]	Prospective and Retrospective
101	33	33%	1 CPC 4 32 CPC 5	12 (described)	12%	36%	Sedation TTM (33 °C)	1 CPC 4 11 CPC 5	c-EEG	Rittenberger et al. [32]	Retrospective
60	17	28%	7 CPC < 4 10 CPC ≥ 4	12 (described)	20%	70%	Sedation TTM	4 CPC ≤ 3 8 CPC ≥ 4	s-EEG	Lettieri et al. [33]	Retrospective
38	7	18%	1 CPC 3 1 CPC 4 5 CPC 5	2 (described)	5%	29%	Sedation TTM (33 °C)	2 CPC 5	c-EEG	Mani et al. [34]	Retrospective
25	NA	NA	NA	3 (described)	12%	NA	NA	NA	c-EEG	Claassen et al. [35]	Retrospective
*Studies with information that allow indirect conclusions to be drawn regarding NCSE*											
106	33	31%	2 CPC 1–2 31 CPC 3–5	4 (inferred*)	4%	12%	Sedation TTM (32–34 °C)	1 CPC 1–2 3 CPC 3–5	s-EEG and c-EEG	Legriel et al. [37]	Prospective observational
95	26	27%	1 CPC 2 1 CPC 3 24 CPC 5	1 (inferred*)	1%	4%	Sedation TTM (33 °C)	1 CPC 2	c-EEG	Rundgren et al. [11]	Prospective observational
51	5	10%	5 CPC 5	1 (inferred*)	2%	20%	Sedation TTM (32–34 °C)	1 CPC 5	s-EEG	Legriel et al. [38]	Prospective observational
127	41	32%	1 CPC 1 2 CPC 2 1 CPC 3 37 CPC 5	5 (inferred*)	3%	12%	Sedation TTM (33 or 36 °C)	2 CPC ≤ 3 3 CPC 5	c-EEG	Backman et al. [36]	Retrospective
288	47	16%	10 CPC 1–2 37 CPC 3–5	14 (inferred*)	5%	30%	Sedation TTM (33 °C)	9 CPC 1–2 5 CPC 3–5	c-EEG	Ruijter et al. [19]	Retrospective
127	41	32%	1 CPC 1 2 CPC 2 1 CPC 3 37 CPC 5	6 (inferred*)	5%	15%	Sedation TTM (33 or 36 °C)	2 CPC ≤ 3 4 CPC 5	c-EEG	Dragancea et al. [14]	Retrospective

*CA* cardiac arrest, *SE* status epilepticus, *NCSE* non-convulsive status epilepticus, *N* number, *NA* not available, *CPC* cerebral performance category, *MCS* minimally conscious state, *TTM* target temperature management, *c-EEG* continuous EEG, *s-EEG* standard EEG

*Number of patients with presumed NCSE as no motor symptoms were described

### Studies directly reporting NCSE

The only study we could find in the literature with the main focus on NCSE detection in adult post-CA patients was performed retrospectively in 2012 [32], where NCSE was reported in 12% (12/101) of the cohort. A 22-channel c-EEG recording was performed at a median of 9 h (range 6–12 h), on 101 resuscitated and sedated patients treated with hypothermia with a mean temperature of 33.9 °C for the first 48 h after CA. NCSE was defined as a continuous electrographic seizure lasting 30 min or greater or recurrent electrographic seizures lasting for over 30 min; GPEDs (old term for GPDs) lasting at least 30 min at a rate ≥ 2.5 Hz, GPEDs lasting at least 30 min at a rate of ≥ 1 Hz with unequivocal evolution in frequency, morphology, or location over time.

NCSE was detected at the onset of the recording in three of the 12 (25%) patients and had a median duration of 5 h. The outcome of the 12 NCSE patients was poor with only one patient surviving with a CPC score of 4 and a modified Ranking scale (mRS) score of 5. One patient regained consciousness but died in-hospital from subsequent multiple organ failure. Information regarding the timing of NCSE detection for these two patients were lacking.

Another retrospective study investigating the awakening in post-hypoxic encephalopathic patients described 20% (12/60) having NCSE (clinically and/or electrical), in a total of 17 SE (five patients showing prominent motor symptoms) [33]. Patients were investigated with 11-channel EEGs recorded for at least 30 min but the time from CA to recording is unknown, as well as if patients were under sedation and TTM. NCSE was defined following the Salzburg criteria [30, 31].

Favorable outcome (Glasgow Outcome Score of > 2/CPC of ≤ 3) was identified in seven patients, four of them with NCSE, one myoclonic and two convulsive SE. In particular, three patients showed a CPC score of 2 and four patients a CPC score of 3. Information about the type of SE for the CPC subgroups was lacking.

NCSE was reported in two other studies investigating the predictors of awakening from SE as the consequence of hypoxic-ischemic encephalopathy [9] and the frequency and timing of epileptic activity on c-EEG in comatose patients following ROSC [34].

The first study consisting of a large prospective and retrospective cohort including 181 patients with 74 patients being investigated prospectively in 2009 revealed SE in 38% of the prospectively examined patients (28/74) with 31% showing myoclonus, 1% presenting with prolonged tonic–clonic seizures, and 5% being in NCSE as confirmed by 14 or 21-channel-EEGs [9]. SE was defined by the occurrence of repetitive, rhythmic (at least 2 Hz), focal or generalized epileptiform discharges, or by periodic or rhythmic waves evolving in shape, amplitude, distribution, and frequency over time, and lasting at least 5 min.

While all but one of the SE patients with motor symptoms died, one of the patients with NCSE remained in a minimally conscious state before dying in hospital and another improved (CPC score 2) [9]. The aim of that study, however, was to investigate predictors of awakening which was the case in six patients surviving SE. In those patients, SE occurred from day 2 to 9 with three showing myoclonus and three with NCSE. The outcome at 6 months of NCSE patients was a CPC of 2 in two patients and a short-term outcome with a CPC of 3 before dying of sepsis in the third.

Another retrospective study published in 2012 [34], examining 38 comatose patients treated with therapeutic hypothermia and monitored with a 16–18 channel c-EEG applied within a median of 14.8 h from arrest (range 7.5–21.3 h). SE was found in 18% (7/38) with 13% (5/38) having motors symptoms and 5% (2/38) with NCSE. SE was defined as seizure activity occurring for > 30 min. All patients with NCSE died during their hospital stay.

Finally, a retrospective study regarding epileptic seizures using a 21-channel c-EEG of critically ill patients described 12% (3/25) of NCSE in the subgroup of resuscitated patients with hypoxic-ischemic encephalopathy [35]. As the main aim of this study was to monitor critical ill patients with c-EEG not limited to resuscitated patients, information regarding the exact timing of EEG following CA and outcome post-CA NCSE patients were lacking. The only information provided was that seizures were detected (clinically or on EEG) in 93% of all patients mainly within 2 days. Unfortunately, a definition of NCSE was not provided.

### Studies with indirect information regarding NCSE

The remaining identified studies assessed the frequency of post-hypoxic-ischemic SE, predictors of specific outcomes, and seizure/SE detection by EEG, but were not explicitly looking at NCSE. Thus NCSE-related information could only be obtained indirectly following analytical exclusion procedures as we detailed above.

A retrospective study in 2015 aimed to assess the outcome following SE emerging from post-hypoxic-ischemic encephalopathy [14] using a simplified eight electrode c-EEG. Electrographically proven SE was described in 32% of the patients (41/127). EEG was started within a median of 7 h (range 5–10 h) following CA in the group where SE followed a discontinuous-burst-suppression pattern and 8 h (range 7–14 h) where SE followed a continuous brain activity. 85% of the SE patients (35/41) showed motor symptoms. Therefore, at least 15% (6/41) showed NCSE assuming that none of the clinically overt SE types transformed into NCSE (e.g., SE presenting with subtle clinical features, such as for example with nystagmus). Since authors described two of the four survivors later having clinical convulsions, we assume that two of the NCSE group survived (CPC of ≤ 3) and four died. The same cohort (41 SE patients of 127 post-CA patients) has been retrospectively studied more recently where EEG was initiated at a median of eight hours from CA (range 6–11 h)[36]. Surprisingly, the authors reported one more patient having convulsions during ICU stay (88% of patients, 36/41), and of consequence one fewer NCSE patient (12%, 5/41) as compared to the cohort described 2015. As reported in 2015, only four of the 41 patients (10%) were short-term survivors, with only one having mild neurologic deficits. Since two of the survivors showed convulsions, we assume that two patients of the NCSE group survived (CPC of ≤ 3) and three died.

Another retrospective study regarding the emergence of generalized periodic discharges (GPDs) in post-CA patients using a 21-electrode c-EEG in 2015 [19] found electrographic SE in 16% of the patients (47/288). Again, information regarding the specific SE types was lacking, but the majority (70%, 33/47) of patients were described having motor symptoms. The latter allowing the assumption that the minority (30%, 14/47) had NCSE [19]. All SE patients except one with motor symptoms had poor short-term outcomes (CPC of 3–5) within 72 h after CA, whereas the majority of presumed NCSE patients (64%, 9/14) had a good outcome (CPC of 1–2). EEG was initiated at a median of 13 h (range 3–24 h) in the “poor outcome” group and at 7 h (range 2–17 h) in the “good outcome” group.

A prospective study revealed SE after ROSC as one of the strongest and independent associations with poor outcome (CPC of 3–5) [37]. Patients were treated with therapeutic hypothermia for 24 h and underwent 8-channel EEG after CA within a median of 14 h after CA (range 6–20 h). SE following hypoxic-ischemic encephalopathy emerged in 31% of patients (33/106) at a median of 39 h. As 88% (29/33) of SE patients showed early myoclonus, we can assume NCSE in 12% (4/33). Only 6% of the SE patients (2/33) survived with good recovery (CPC of ≤ 3) after 1 year with one having early myoclonus and another without motor symptoms.

Similar SE frequency (27%, 26/95) was reported in a prospective study of 95 CA patients treated with hypothermia and monitored with a 5-channel c-EEG [11]. EEG was started at a median of 8 h after CA (range 5–14 h) revealing SE based on EEG features: one developing from a burst-suppression and the other emerging from continuous background activity. Motor symptoms were seen in 25 of 26 SE patients, ranging from discrete facial myoclonic twitches to generalized tonic–clonic convulsions. Only one patient presented NCSE and regained consciousness (defined as at least reproducible simple movement on command) 2 days later and recovered to a CPC score of 2.

In another prospective study aiming to determine whether bipolar 8-channel EEG detects SE otherwise masked by the use of neuromuscular blockades in comatose CA survivors receiving therapeutic hypothermia, SE was uncovered in 10% (5/51) [38]. Motor symptoms, such as continuous myoclonus, were seen in four of the five patients, occurring only after rewarming in two, and before initiation of hypothermia in the other two. EEG was performed at a median of 12 h (range 7–14 h) following CA. No patient survived.

## Discussion

Our review of the literature identified 11 studies with different designs, reporting heterogenic cohorts, and mostly providing limited information regarding NCSE. We could not identify any studies reporting early EEG monitoring to detect NCSE in resuscitated adult patients remaining in coma during the first hours after ROSC (i.e., < 7.5 h) and prior to the initiation of targeted temperature management and sedation. Remarkably, considering only those patients described to present a CPC score of 1 and 2, NCSE following ROSC was associated with favorable outcome in every fourth patient indicating that equating NCSE in the early stage post-ROSC with poor prognosis is unjustified. In addition, it remains unclear to what extent earlier detection and treatment of NCSE may have influenced patients' outcome.

With the exception of the retrospective studies by Rittenberger et al. in 2012 [32] using 22-channel c-EEGs and providing the frequency of NCSE in post-CA encephalopathic patients, and the one by Lettieri et al. in 2017 [33] using 11-channel EEGs, studies were not specifically designed to investigate the emergence of NCSE. Moreover, EEG monitoring was usually not performed prior to targeted temperature management and sedation, and sometimes used simplified (i.e., limited) EEG. Remarkably, the proportion of NCSE patients was highest (12% [32] and 20% [33]) in those two studies [32, 33] as compared to the other nine investigations [9, 11, 14, 19, 34–38], and lower (ranging from 1 to 5%) in studies not explicitly reporting NCSE, but SE with motor symptoms [11, 14, 19, 36–38].

Three of those studies [9, 32, 35] have been already reported in a previous review about treatment and recognition of NCSE in the ICU where the key importance of c-EEG monitoring in this setting was highlighted, particularly in those patients with “unexplained (or perhaps apparently explained) coma” [39].

These findings strongly suggest that NCSE can be frequently missed, remains overlooked, untreated, and misjudged in these critically ill and comatose patients.

Moreover, the absence of evidence in literature of NCSE in the first and early hours (i.e., < 7.5 h) after ROSC without or after the weaning of initial anesthesia that might have been established by the emergency medical teams does not equal evidence of absence of early NCSE.

The question on whether a persistent coma after ROSC may be explained by an early NCSE does therefore remain unanswered and should be the center of upcoming studies in the context of neurologic course and outcome in resuscitated patients. That would need a first EEG at ICU admission, that should be obviously postponed in favor of the initially necessary and more vital support interventions.

In the study from Rittenberger et al. [32], NCSE was detected at the onset of the EEG recording in 25% of NCSE patients, indicating that there might have been a substantial proportion of patients in NCSE shortly after ROSC; whereas in the one from Backman et al. [36] no SE was detected at EEG onset, confirming others’ findings showing how epileptiform activity increased over time [40].

Detailed information regarding specific long-term outcomes were frequently missing and none of the studies reporting outcomes following NCSE after ROSC addressed and/or excluded a self-fulfilling prophecy that is likely to have resulted from withdrawal or withholding of care once physicians uncovered NCSE. The latter cannot be excluded with certainty without a priori blinding the physicians to NCSE.

The limited quality of current data do not allow any firm conclusions regarding the direct effects of NCSE on short- or long-term outcomes. The presence of SE post-CA was associated with an increased overall, short- and long-term mortality, while SE post-CA during the first EEG (but always performed after TTM and at least 24 h without sedation) was only associated with an increased short-term mortality. The findings are interesting in themselves, but we cannot extend them to NCSE post-CA, since authors did not distinguish between NCSE and SE with motor symptoms [41].

It seems, however, likely that NCSE is frequently associated with good outcome as compared to SE with motor symptoms [19, 37], but the few studies and the low quality of data do also not allow any further conclusion in this context. Moreover, it is unclear from those studies whether conditions with alternating NCSE and SE with motor symptoms can co-exist in the same patient indicating that seizure duration may be even longer as clinically suspected by overt motor symptoms and have specific effects on outcome. Finally, we cannot exclude that treating resuscitated patients with deep sedation and muscle relaxants to control myoclonus or subtle motor symptoms including shivering, would transform SE with motor symptoms into NCSE potentially leading to even higher numbers of NCSE than reported.

Overall, the EEGs were rarely performed early on, across studies (the earliest was at a median of 7.5 h; Fig. 1), and in different time windows across and among studies; sometimes the time EEG was started was not even reported. Many studies performed EEG with simplified montages, not allowing detailed analysis of the electrical cortical activity. Moreover, EEG, even if performed early, was always performed in patients already under hypothermia and sedation, both having well-known potential antiseizure effects. Therapeutic hypothermia per se has antiseizure effects [42] and often requires the administration of anesthetics, which may exert further powerful antiseizure effects. Moreover, hypothermia can increase blood concentrations of propofol [43] and its anticonvulsant effects. None of the studies directly reporting NCSE reported the effects of the sedation, a fact that represents an important limitation.

Most importantly, we cannot exclude that the new guidelines on temperature control [6] will have an impact on the detection rate of NCSE in future studies. In scenarios with initial anesthesia which is not stopped before an EEG is performed during the initial clinical assessment after ROSC, the question whether such ongoing anesthesia within the early hours has the ability to successfully terminate seizures in case of undetected NCSE despite not strictly following the treatment guidelines for SE, including the establishment of an EEG burst-suppression pattern for 24–48 h as recommended [44], remains unanswered. A recent trial showing no benefit of aggressive antiseizure treatment of any rhythmic and periodic discharges in comatose post-CA patients still does not answer this question, since the targeted patients did not exclusively have SE as defined accordingly to the guidelines [21, 29].

The concept of a “three-dimensional” biological continuum well shows the complexity in the interrelation between structural brain damage, epileptic activity, and the degree of coma, and how an excessive epileptic activity may play a role in the worsening of the brain damage itself [45].

Despite these vague conclusions, the current data should prompt clinicians to be cautious when it comes to outcome prediction in NCSE patients following ROSC, as many questions remain unanswered, such as the following:

- Can aggressive and rapid antiseizure treatment in the context of early NCSE following ROSC optimize outcome?
- Is sedation, when not titrated to produce a predefined EEG coma pattern adequately terminating clinically missed NCSE?
- How early does NCSE emerge following ROSC in patients not regaining consciousness?
- Will the detection of NCSE post-CA increase following the new temperature control guidelines that may reduce the need of anesthesia?
- When and how close should we monitor comatose CA survivors? Does it always have to be at ICU admission and continuously?
- Do we need to monitor our patients with a higher spatial resolution using at least 21–25 scalp electrodes?

## Conclusion

Studies on NCSE after ROSC in adult patients are rare and mostly unsystematic. Favorable outcomes have been reported in every fourth patient suggesting that outcome after NCSE is not invariably poor. The data compiled suggest that with heightened awareness and early systematic EEG-driven screening for NCSE in patients remaining comatose after ROSC and after initial anesthesia had been weaned, the detection rate of NCSE will increase.

Further studies are urgently needed to clarify the effect of post-CA NCSE on outcome and if increased NCSE detection, even at the very early stages following ROSC, and subsequent aggressive antiseizure treatment may influence outcome. In the meantime, clinicians should avoid equating NCSE after ROSC with an inevitable poor prognosis.
